# Differences and Similarities in the Mechanisms and Clinical Expression of Bradykinin-Mediated vs. Mast Cell–Mediated Angioedema

**DOI:** 10.1007/s12016-021-08841-w

**Published:** 2021-02-03

**Authors:** Marcus Maurer, Markus Magerl

**Affiliations:** Dermatological Allergology, Allergie-Centrum-Charité, Department of Dermatology and Allergy, Charité – Universitätsmedizin Berlin, corporate member of Freie Universität Berlin, Humboldt-Universität zu Berlin, and Berlin Institute of Health, Berlin, Germany

**Keywords:** Angioedema, Bradykinin, Mast cell, Hereditary angioedema, Urticaria

## Abstract

Angioedema (AE), transient localized swelling due to extravasated fluid, is commonly classified as mast cell mediator-induced, bradykinin-mediated or of unknown cause. AE often occurs more than once, and it is these recurrent forms of AE that are challenging for patients and physicians, and they are the ones we focus on and refer to as AE in this review. Since effective treatment depends on the causative mediator, reliable and early diagnosis is essential. Although their clinical presentations bear similarities, many forms of angioedema exhibit specific patterns of clinical appearance or disease history that may aid in diagnosis. Here, we describe the most common differences and similarities in the mechanisms and clinical features of bradykinin-mediated and mast cell mediator-induced types of angioedema. We first provide an overview of the diseases that manifest with mast cell mediator-induced versus bradykinin-mediated angioedema as well as their respective underlying pathogenesis. We then compare these diseases for key clinical features, including angioedema location, course and duration of swelling, attack frequency, prevalence and relevance of prodromal signs and symptoms, triggers of angioedema attacks, and other signs and symptoms including wheals, age of onset, and duration. Our review and comparison of the clinical profiles of different types of angioedema incorporate our own clinical experience as well as published information. Our aim is to highlight that mast cell mediator-induced and bradykinin-mediated angioedema types share common features but are different in many aspects. Knowledge of the differences in underlying pathomechanisms and clinical profiles between different types of angioedema can help with the diagnostic approach in affected patients and facilitate targeted and effective treatment.

## Introduction

Angioedema (AE) is defined as a transient localized swelling due to extravasated fluid. In clinical terms, AE is often a chronic disease with swellings that occur more than once rather than a solitary episode of swelling. The latter are mostly allergic reactions, for example, in the context of anaphylaxis due to allergen exposure. Here, we focus on AE forms that manifest with recurrent swellings, as they are the ones that are challenging for patients and physicians, and we use the term AE to refer to these recurrent forms of AE.

AE virtually always involves the lower layers of the skin (dermis) and the highly vascular subcutis or the mucous membranes, lasting from a few hours up to several days. In most cases, angioedema occurs spontaneously, but may be induced by various “triggers” [1]. In patients with AE, i.e., repeated angioedema attacks for longer than 6 weeks, swelling episodes are separated from each other by symptom-free intervals of a variable duration. Angioedema of the skin is non-pitting, frequently asymmetric, mostly non-pruritic, and can be rather painful. Depending on its location, AE can cause disfigurement, physical dysfunctionality, pain, or obstruction of airways, thereby impairing patients’ quality of life.

AE is commonly classified as *mast cell mediator-induced AE* (MCM-AE), *bradykinin (BK)-mediated AE* (BK-AE), and *AE of unknown cause*, based on the underlying pathogenesis. Here, we describe differences and similarities in the mechanisms and clinical features of MCM-AE and BK-AE.

## Forms of Mast Cell-Mediated AE and Underlying Pathogenesis

### Diseases that Manifest with MCM-AE

Chronic urticaria is by far the most common disease that manifests with MCM-mediated AE [2]. AE in patients with chronic urticaria more often occurs in those with chronic spontaneous urticaria (CSU) than chronic inducible urticaria (CIndU). CSU is a common disease with a prevalence of up to 1.4% in the general population [3]. It is defined by the recurrent occurrence of wheals, angioedema, or both. AE, with or without wheals, occurs in up to 70% of patients with CSU [4, 5], and around 10% of CSU patients only have AE without wheals [6]. In CSU, AE is linked to prolonged disease duration and high burden of disease [7]. However, AE is less common in patients with CIndU (such as cold or cholinergic urticaria). In a recent study, 46% of patients with cholinergic urticaria (CholU), a CIndU characterized by perspiration-induced signs and symptoms, had AE [8]. Cholinergic urticaria (CholU) patients with AE were significantly more likely to have a prolonged disease duration and to experience extracutaneous signs and symptoms than CholU patients without AE. In symptomatic dermographism, a CIndU with scratching-induced signs and symptoms, one in five patients reports AE, mostly localized to the eyelids, legs, and hands [9]. The rates and relevance of AE in other CIndUs are less well characterized. Outside of chronic urticaria MCM-AE may also occur in the context of anaphylaxis.

### The Pathogenesis of MCM-AE

Mast cells are organ-resident cells of the innate and adaptive immune system [10, 11]. Their main physiological role is to provide a first line of defense against pathogens and other environmental threats [12–14]. In the skin, mast cells are co-localized with sensory nerves and small blood vessels. Mast cell activation and the subsequent release of preformed mediators, including histamine and proteases, induce sensory nerve stimulation (pruritus, burning sensation, pain), vasodilatation (erythema), increased plasma extravasation (edema) and the recruitment of eosinophils, basophils, and other immune cells (cellular infiltrate). Mast cell activation is a complex process that is initiated by a large array of signals, many of which act via specific mast cell surface receptors. Prominent mast cell-activating receptors include the high affinity IgE receptor FceRI, receptors for the complement components C3a and C5a, and the Mas-related G-protein coupled receptor member X2 (MRGPRX2) [15].

In chronic urticaria, angioedema and wheals are induced by the degranulation of mast cells and the effects of the mediators they release, mainly histamine. Histamine acts on H1 receptors located on vascular endothelial cells to cause extravasation (wheals and angioedema) and on sensory nerves, resulting in neurogenic flare (erythema) and pruritus. Effects on H2 and H4 receptors may also be involved. Several other mediators of the mast cell, like leukotrienes, or the platelet activating factor can cause similar responses to histamine, and thereby amplify and prolong the inflammatory process. Importantly, the recruitment of inflammatory cells to skin sites of mast cell activation and degranulation, in most forms of chronic urticaria, contributes to the features of wheals and angioedema and primes the skin for subsequent whealing and angioedema development [16, 17].

In terms of the signals that contribute to skin mast cell activation and degranulation in chronic urticaria and, therefore, angioedema and wheal development, true mast cell degranulators need to be distinguished from mast cell modulators. The first lead to degranulation and are therefore essential for the release of proinflammatory mast cell mediators and subsequent development of angioedema. Mast cell modulators alter the skin mast cells threshold, and therefore augment their response to mast cell degranulators [18].

Two different classes of autoimmune antibodies lead to skin mast cell degranulation in CSU [19]: (a) Specific IgE antibodies against autoantigens [20–22] and (b) IgG and IgM autoantibodies against IgE or the alpha subunit of the IgE receptor [23, 24]. Infection-associated signals, food components, and neuropeptides are seen today, as relevant mast cell modulators. Mental stress is also regarded as a relevant modulator of CSU disease activity in many patients. Neuropeptides that are released during stress reactions have mast cell-modulating effects.

## Forms of BK-Mediated AE and Underlying Pathogenesis

### Diseases that Manifest with BK-AE

BK-AE occurs in hereditary angioedema (HAE) with or without C1 inhibitor (C1-INH) deficiency, AE due to acquired C1-INH deficiency (C1-INH-AAE), and angiotensin-converting enzyme inhibitor (ACEi)-associated AE. In general, most of these diseases present with AE in the absence of wheals are considered rare diseases, except ACEi-associated AE (due to a very high global usage of ACEi) [1, 25].

Hereditary angioedema due to C1-INH deficiency (C1-INH-HAE): This is the most common form of HAE and results from a quantitative (Type 1, approximately 85% of patients) or functional (Type 2, approx. 15%) deficiency of C1-INH, the main plasma serine-protease inhibitor of the BK-forming system. Both type 1 and 2 C1-INH-HAE are caused by mutations in the C1-INH gene, SERPING1, and are inherited as autosomal dominant trait. However, the percentage of spontaneous mutations (new mutations) is high at approximately 20%. The minimal prevalence of HAE is 1:67,000 in the general population, without evidence of any gender, ethnic, or racial differences [1, 25–27].

Hereditary angioedema with normal C1-INH (nC1-INH-HAE): The clinical appearance of nC1-INH-HAE resembles that of C1-INH-HAE; however, most patients are female, and their C1-INH and plasma complement levels are normal. As of now, mutations in 5 genes were found to be pathogenic: Factor XII (FXII) [28], plasminogen (PLG) [29], angiopoietin-1 (ANGPT1) [30], kininogen-1 (KNG1) [31], and myoferlin (MYOF) [32]. By now, more than 400 patients with FXII-HAE and more than 100 patients with PLG-HAE have been reported. Patients with ANGPT1-HAE, KNG1-HAE, and MYOF-HAE were only described in few individuals and families. FXII-HAE, PLG-HAE, and KNG1-HAE are considered to be bradykinin-mediated forms of AE, with excess formation of BK, whereas ANGPT1-HAE and MYOF-HAE are held to be due to vascular endothelial receptor modulation, respectively, VEGF signal transduction. Women are strikingly overrepresented amongst patients, and estrogen-sensitivity has been described very frequently in FXII-HAE and frequently in PLG-HAE [33]. It should be kept in mind that nC1-INH-HAE is an umbrella term for a variety of AE diseases, many of which are extremely rare, and clinical data are lacking at the present time. Therefore, the description and characterization of their clinical features as one disease are oversimplied.

AE due to acquired C1-INH deficiency (C1-INH-AAE): C1-INH deficiency can also be acquired. It occurs due to increased catabolism of C1-INH, when C1-INH degradation outperforms the synthesis of new C1-INH. Patients with C1-INH-AAE often have an underlying disease such as a lymphoproliferative disorder that leads to continuous activation of the classic complement pathway with consequent depletion of C1-INH. Some patients may also develop autoantibodies to C1-INH protein, which interferes with its level and activity. Like C1-INH-HAE, AE in patients with C1-INH-AAE has elevated plasma BK levels [34, 35].

Angiotensin converting enzyme (ACE) inhibitor-associated angioedema (ACEi-AE): ACE is a protease that cleaves BK. When ACE is inhibited, BK degradation is impaired, which facilitates the development of AE. Indeed, patients with ACEi-AE have been reported to have increased plasma BK levels. Dipeptidyl peptidase-4 inhibitors (DPPIV) or neprilysin (neutral endopeptidase) inhibitors used in the treatment of hypertension can increase the propensity of drug-induced angioedema significantly when combined with ACEi [36–38].

## The Pathogenesis of BK-Mediated AE

BK is a vasoactive nonapeptide that promotes vasodilatation via the BK B2 receptor [39, 40]. The BK-forming cascade in the plasma, i.e., the contact system and kallikrein-kinin pathways, consists of activated factor XII(a), (pre)kallikrein (PK), kallikrein (PKa), and high-molecular-weight kininogen (HK). This cascade is initiated when FXII and PK are activated and form FXIIa and PKa, which cleaves HK to release BK [41, 42]. C1-INH regulates this cascade, by binding to the active sites of factor XIIa and kallikrein, thereby inactivating them. BK is degraded by ACE (kininase 2), carboxypeptidase N, neutral endopeptidase, dipeptidyl peptidase IV, and aminopeptidase P, resulting in extremely short half-life of 17 s [43, 44]. Both increased production and inhibited degradation can cause excess of BK and subsequent angioedema.

### Differences and Similarities of MCM-AE and BK-AE

MCM-AE and BK-AE are different in many aspects but also share common features (Table 1). Since the prognosis and treatment in patients with MCM-AE and BK-AE are fundamentally different, it is important to know how to tell the two apart [45]. Knowledge of these differences can help with the diagnosis and treatment of patients with AE.

**Table 1:** Overview of differences and similarities between MCM-AE and BK-AE

	C1-INH-HAE	nC1-INH-HAE	C1-INH-AAE	ACEi/RAAS	AsU/CsU
Recurrent laryngeal angioedema	++	++	++	++	-
Predominantly recurrent isolated tongue swelling	-	+ (PLG)	-	++	+
Wheals present/history of recurrent wheals	-	-	-	-	++
Recurrent painful abdominal attacks	++	+	++		-
Tongue/lip swellings, usually unilateral at onset				+	+
Head/neck swelling onset usually early morning hours					+
Prodromal symptoms	++			-	-
Decade of symptom onset	0-2	0-4	>6	>5	>0
Family history of recurrent angioedema and/or abdominal pain/deaths by asphyxiation	++	++	-	-	-
Mainly females in family history	-	++			
Estrogen sensitivity	+	++	+		
Angioedema repeatedly triggered by NSAID's	-	-	-	-	+

+: frequent or typical, ++: very frequent or very typical, -: rare or unusual, blank: indetermined or unknown

*C1-INH-HAE* HAE with C1-INH deficiency, *nC1-INH-HAE* HAE with normal C1-INH, *C1-INH-AAE* angioedema due to acquired C1-INH deficiency, *ACEi / RAAS* angioedema due to ACE-inhibitors and the renin–angiotensin–aldosterone system, *AsU/CsU* acute/chronic spontaneous urticarial, *PLG* plasminogen-gene mutation, *NSAID* nonsteroidal anti-inflammatory drugs

## Differences and Similarities in Angioedema Features of MCM-AE and BK-AE

### Angioedema Location

All types of AE, MCM-AE, and BK-AE primarily involve the cutis/mucosa and subcutis/submucosa, affect several organs, and manifest in many locations of the body. The skin of the face, including the lips and the oral mucous membranes, is common location of swellings in all types of AE. MCM-AE and BK-AE show different patterns regarding their preferred localization, but there are also differences between the different types of BK-AE.

Involvement of the tongue, including isolated tongue swellings, is frequent in MCM-AE but also in most forms of nC1-INH-HAE, especially PLG-HAE, and in ACEi-AE. Isolated AE of the tongue is relatively rare in C1-INH-HAE and C1-INH-AAE [33, 46–48]. Clinical experience suggests that there is benefit in asking patients whether lip or tongue swellings begin unilaterally, as a unilateral onset of lip or tongue angioedema is more likely to occur in ACEi-AE and MCM-AE rather than in C1-INH-HAE or C1-INH-AAE.

Involvement of the larynx and oropharynx with the risk of asphyxiation and fatal outcome is predominantly seen in BK-AE, most of all in ACEi-AE and C1-INH-HAE. Up to 50% of patients with C1-INH-HAE experience laryngeal swellings, that if not treated, can lead to death by asphyxiation [49–52]. In MCM-AE, shortness of breath due to swellings of the upper airways (pharynx, tongue) can be seen in some cases, albeit very rarely, especially in chronic urticaria patients with MCM-AE. Laryngeal swellings with a fatal outcome have never been reported in chronic urticaria patients.

AE of the earlobes points to MCM-AE due to chronic urticaria. This location is usually described with redness and warming of the skin, along with itch or a burning sensation. Thus, AE of the earlobes, in patients with chronic urticaria, usually shares features of urticarial wheals. Ear swellings have also been reported in patients with FXII-HAE but appear to be rare in BK-AE [33].

Almost all patients with C1-INH-HAE experience swellings of the extremities, and by far the most swelling episodes are located in these skin regions [53]. Angioedema of the skin of the limbs occurs in MCM-AE as well as in BK-AE. Peripheral skin swellings, especially angioedema of the hands and feet, are typical for C1-INH-HAE. Swellings of the extremities are less common in patients with other types of BK-AE such as nC1-INH-HAE, and they are very rare in PLG-HAE and ACEi-AE [54]. In contrast, the skin of the limbs is often affected and is the second most frequent localization of swellings in patients with MCM-AE, second only to the skin of the head and neck.

Abdominal attacks, i.e., painful episodes of angioedema of the gut walls, are seen in almost all patients with C1-INH-HAE and are associated with severe pains lasting for many hours to several days [53]. In nC1-INH-HAE, the occurrence of abdominal angioedema attacks varies between the different types and appears to be more frequent in FXII-HAE and rather rare in PLG-HAE [33]. A small number of reports describe abdominal swellings as a rare and unusual feature of ACEi-AE [47, 55, 56]. Abdominal angioedema attacks are usually not seen in patients with MCM-AE, but a small rate of patients with CSU reports mild or moderate abdominal complaints [54].

The genitals are affected in most patients with BK-AE (i.e., C1-INH-HAE and C1-INH-AAE), although angioedema of the genitals is rare compared with other skin sites. In all other forms of AE, genital angioedema is infrequent.

### Course and Duration of Swellings

In MCM-AE, the time between the onset of angioedema, often a tingling sensation, and the maximum of the swelling can be less than 30 min, but usually it takes 1 to 4 h. After the angioedema has fully developed, it slowly resolves and subsides within 12 to 24 h, in most cases. AE, in patients with BK-AE, usually develops at a slower pace, over the course of several hours [26, 57, 58]. However, some swellings develop rapidly, especially abdominal and laryngeal attacks. The reasons remain unclear but may be explained by the fact that the key symptoms, pain and difficulty in breathing, respectively, reach their maximum effect earlier than those of the peripheral swellings. In most patients with C1-INH-HAE or C1-INH-AAE, swellings progress for 6 to 24 h from the onset to reaching the maximum. Untreated C1-INH-HAE attacks rarely last less than 36 h but sometimes longer than 6 days, if left untreated. Reliable newer data are hard to find, as patients are advised to treat their attacks early and often do. There is very little information on the course of attacks in patients with nC1-INH-HAE. In patients with ACEi-AE, swellings develop usually within a few hours, typically 6 to 12 h; however, faster or slower progress to maximum has been described in many cases [59, 60]. Of note, the development of the swelling is not necessarily linear over time, but can progress more rapidly after a slow onset. Usually, swellings in patients with ACEi-AE resolve within 48 to 72 h.

### Frequency of Swellings

The frequency of swellings underlies wide intraindividual and interindividual variations. In all types of AE, affected patients can develop swellings as often as several times per week, whereas others can go months and sometimes years without any attacks, although this is very rare. On average, patients with nC1-INH-HAE report less frequent episodes of swellings than other forms of BK-AE [61]. The median number of attacks in nC1-INH-HAE was reported to be approximately 5 per year, whereas C1-INH-HAE and C1-INH-AAE patients were reported to suffer from approximately 10 attacks per year [46, 61–64]. Patients with MCM-AE due to CSU are held to show the highest average frequency of swelling episodes. Of note, disease activity including rates of attacks, with the help of suitable tools such as the angioedema activity score (AAS, [65, 66]), has not yet been assessed and compared prospectively across patient population with MCM-AE and BK-AE.

## Differences and Similarities In Clinical Features of Diseases That Manifest with MCM-AE and BK-AE

### Prodromal Signs and Symptoms

AE prodromes are unique to BK-AE, specifically C1-INH-HAE. In up to 80% of patients with C1-INH-HAE, angioedema attacks are often, but not always, heralded by prodromal signs or symptoms [67–70]. Common prodromal signs and symptoms include typical *erythema marginatum* pain, fatigue, or nausea. Prodromes occur mostly within 6 h before an attack and must be strictly differentiated from the symptoms of the emerging actual attack. Prodromes have not been commonly described in other forms of AE, with the exception of unspecific prodromes in a small number of patients with FXII-HAE [71].

### Triggers of Angioedema Attacks

Swellings in all types of AE have been reported to occur in response to inciting factors (triggers). What has to be kept in mind is that the assessment of the prevalence and relevance of triggers for selling attacks is difficult, as it relies on the patients’ perception of the relation between triggers and attacks. Triggers are often only reported, when patients suspect them to be linked to inducing swellings. Furthermore, many triggers such as stress are difficult to authenticate.

Emotional distress is held to be a relevant trigger of angioedema attacks in 21–23% of patients with C1-INH-HAE, which makes it the most frequent trigger in this form of AE [67, 72]. In most types of nC1-INH-HAE the role of stress as a trigger remains unclear, except in FXII-HAE, where, in two large patient populations, stress was found to be a trigger in approximately 50% of the patients [71, 73]. MCM-AE, in patients with CSU, is widely held to be linked to emotional stress, and CSU is known to worsen with increased levels of stress [74]. However, the prevalence and relevance of stress as a trigger of angioedema episodes have not yet been well established. Physical exertion, acute viral infections, and foods are known triggers in subpopulations of patients with C1-INH-HAE and MCM-AE. A frequent trigger of BK-AE is mechanical trauma, such as pressure, vibrations, surgical manipulations (i.e., oral mucosal pressure during dental procedures), and physical contact during recreational sport [67, 72]. This kind of “reactive angioedema” at the site of preceding mechanical trauma seems to be specific for C1-INH-HAE and FXII-HAE as well as for symptomatic dermographism and delayed pressure urticaria, two forms of CIndU [72, 73, 75].

Drugs are frequent triggers of angioedema episodes, across all forms of both MCM-AE and BK-AE. ACE-inhibitors, the causative agents in ACEi-AE, are known to increase attack rates in patients with C1-INH-HAE, C1-INH-AAE, and FXII-HAE, but not MCM-AE [76, 77]. Estrogen treatment also drives disease activity in patients with C1-INH-HAE, and even more in nC1-INH-HAE. Patients with BK-AE should, therefore, avoid estrogen medication (i.e., oral contraceptives). ACE-inhibitors and thrombolytic treatments (with recombinant tissue plasminogen activators—rtPA) may also induce oro-phayngeal AE due BK dysregulation. In MCM-AE patients with CSU, estrogens are only sporadically reported as triggers, although female patients sometimes see their menstrual cycle linked to disease activity.

MCM-AE is a frequent and well-known side effect of nonsteroidal anti-inflammatory drugs (NSAIDs), particularly in patients with CSU. Clinical experience shows that the relevance of NSAIDs as triggers of swellings in patients with MCM-AE is widely restricted to their use as pain killers, antipyretics, and anti-inflammatory agents, i.e., in high doses, whereas low doses of NSAR, for example, acetylsalicylic acid used as an antithrombotic agent, rarely trigger swellings. NSAIDs do not trigger BK-mediated AE, but can, of course, induce MCM-mediated angioedema in patients with BK-AE.

### Signs and Symptoms Linked to AE Including Wheals

MCM-AE, in most affected patients, is due to urticaria. At least two thirds of urticaria patients with MCM-AE also develop recurrent wheals (hives), whereas recurrent wheals (urticarial skin rash) are not a typical feature of any BK-AE type. Therefore, as a general rule, recurrent wheals with AE indicate MCM-AE due to chronic urticaria, most commonly CSU. Neverthless, the absence of wheals does not rule out MCM-AE, as about 10% of patients with CSU, develop only AE and never have wheals. Urticaria is one of the most common dermatological diseases [78]. Most patients experience urticaria at least once in their lives, mostly acute spontaneous urticaria, which remits within days and weeks. Patients with BK-AE are equally susceptible to contracting urticaria as the general population. This has been shown in a recent publication of Rasmussen and coworkers, where a total of 22 patients (25%) with C1-INH-HAE reported former episodes of urticaria with wheals [70]. Therefore, the occurrence of wheals in the history or physical examination does not rule out BK-AE as a diagnosis.

Recurrent subcutaneous hemorrhage (petechiae) at sites of skin swellings occurring 1 or 2 days after the onset of the swellings was described in some FXII-HAE patients. The hemorrhages were limited to the site of the skin swelling [46, 71, 79]. This phenomenon does not happen in patients with MCM-AE.

## Differences and Similarities in the Onset and Duration of Disease in MCM-AE and BK-AE

### Onset of Disease

The age of onset of BK-AE varies widely. Patients with C1-INH-HAE are born with this trait, but they usually become symptomatic in the first two decades of life, on average around the age of 12 years. Patients with nC1-INH-HAE often start to develop swellings in the third decade of life, but the reported variations are very large. Patients with ACEi-AE or C1-INH-AAE are, on average, around 60 years of age when they first develop swellings. In contrast, MCM-AE can start at any age, but most patients are in their 30s or 40s when they first develop swelling episodes [47, 61, 80].

### Duration of Disease

HAE as a genetic disease is a life-long condition. C1-INH-AAE persists throughout life or until therapeutic or spontaneous remission of the underlying disease. There is no data on ACEi-AE, but usually the establishment of the diagnosis goes along with the discontinuation of the ACE inhibitor. There is also little data on MCM-AE. Data from patients with CSU show that most patients are affected by angioedema for more than 1 year, 5 to 7 years on average, with a considerable number of patients affected for longer periods of time [81].

## Conclusions, Open Questions and the Need for Further Studies

Here, we reviewed the similarities and differences of the mechanisms and clinical expression of BK-AE and MCM-AE. Knowledge of these differences, together with differences in laboratory markers, which are reviewed elsewhere [82], can help to reduce the rates of misdiagnosis and delayed diagnosis.

We are still lacking information on some important features and characteristics of AE, both for MCM-AE, BK-AE. Little is known, for example, about the histological features of BK-AE, the kinetics of cellular infiltration, and the cellular composition of the infiltrate. Other features are well characterized for both types of AE but are difficult to compare, based on the available information. Studies are available on MCM-AE and BK-AE and its association with other comorbidities, burden of disease, impact on sleep, and social, mental, and sexual health. More studies are required to compare these entities, controlling patients for age, gender, genetic background, and other important confounders, by using tools that are suitable for all AE patients such as the angioedema activity score (AAS) or the angioedema control test (AECT) [83, 84]). Ideally, such studies should be performed in a global setting and by professional networks, such as that of angioedema centers of reference and excellence (ACARE, [85]).

One of the biggest challenges in AE management in clinical practice is AE of unknown cause, i.e., AE in patients who do not fulfill the diagnostic criteria of known MCM-AE or BK-AE types. AE profiling by international guidelines, established AE type-specific biomarkers, may help to guide the diagnostic workup and advance the quality of treatments for affected patients.

## Abbreviations

ACEi: Angiotensin converting enzyme inhibitors
AE: Angioedema
ANGPT1-HAE: HAE with angiopoietin mutation
AsU/CsU: Acute/chronic spontaneous urticaria
BK-AE: Bradykinin-induced angioedema
BK: Bradykinin
CSU: Chronic spontaneous urticaria
CIndU: Chronic inducible urticaria
C1-INH: C1 Esterase Inhibitor
C1-INH-AAE: Angioedema due to acquired C1-INH deficiency
C1-INH-HAE: HAE with C1-INH deficiency
cHK: Cleaved HK
FXII: Coagulation Factor 12 (Hageman factor)
FXII-HAE: HAE with Factor XII mutation
HAE: Hereditary angioedema
HK: High molecular kininogen
IgE: Immunoglobulin E
KNG1-HAE: HAE with kininogen 1 mutation
MCM-AE: Mast cell mediator-induced angioedema
MYOF-HAE: HAE with myoferlin mutation
nC1-INH-HAE: HAE with normal C1-INH
PK: PreKallikrein (Fletcher factor)
PKa: Plasma kallikrein
PLG-HAE: HAE with plasminogen mutation
RAAS: Renin–angiotensin–aldosterone system
tPA: Tissue plasminogen activator
U-HAE: HAE with unknown mutation and unknown cause
VEGF: Vascular endothelial growth factor
